# A proficiency assessment of integrating machine learning (ML) schemes on Lahore water ensemble

**DOI:** 10.1038/s41598-023-32280-6

**Published:** 2023-03-29

**Authors:** Nazish Shahid

**Affiliations:** Department of Mathematics, Forman Christian College (A Chartered University), Lahore, Pakistan

**Keywords:** Climate sciences, Mathematics and computing

## Abstract

A synthesis of statistical inference and machine learning (ML) tools has been employed to establish a comprehensive insight of a coarse data. Water components’ data for 16 central distributing locations of Lahore, the capital of second most populated province of Pakistan, has been analyzed to gauge current water stature of the city. Moreover, a classification of surplus-response variables through tolerance manipulation was incorporated to debrief dimension aspect of the data. By the same token, the influence of supererogatory variables’ renouncement through identification of clustering movement of constituents is inquired. The approach of building a spectrum of colluding results through application of comparable methods has been experimented. To test the propriety of each statistical method prior to its execution on a huge data, a faction of ML schemes have been proposed. The supervised learning tools pca, factoran and clusterdata were implemented to establish an elemental character of water at elected locations. A location ‘LAH-13’ was highlighted for containing an out of normal range Total Dissolved Solids (TDS) concentration in the water. The classification of lower and higher variability parameters carried out by Sample Mean (XBAR) control identified a set of least correlated variables pH, As, Total Coliforms and E. Coli. The analysis provided four locations LAH-06, LAH-10, LAH-13 and LAH-14 for extreme concentration propensity. An execution of factoran demonstrated that specific tolerance of independent variability ‘0.005’ could be employed to reduce dimension of a system without loss of fundamental data information. A higher value of cophenetic coefficient, c = 0.9582 provided the validation for an accurate cluster division of similar characteristics’ variables. The current approach of mutually validating ML and SA (statistical analysis) schemes will assist in preparing the groundwork for state of the art analysis (SOTA) analysis. The advantage of our approach can be examined through the fact that the related SOTA will further refine the predictive precision between two comparable methods, unlike the SOTA analysis between two random ML methods. Conclusively, this study featured the locations LAH-03, LAH-06, LAH-12, LAH-13, LAH-14 and LAH-15 with compromised water quality in the region.

## Introduction

The gradual rise of climatic pollution alarmingly points to an urgent need of acquiring high-tech skills to assess and manipulate the inferential bearings in accordance with global environmental regulations. Water resources pollution is one of the main factors contributing to the global environmental decline. The adverse effects^20,25^ of contaminated water on human health are reflected through a general upsurge in water-borne diseases. It is imperative to be cognizant of statistical composition of water structure to determine its fit for domestic and industrial usage. Multiple scholarly efforts have been conducted to investigate the quality of water through various numerical, analytical and experimental endeavours^3,12,14,18,27,33^.

To determine the stature of water, a meticulous characteristic evaluation of constituents needs to be executed through physiological, biological and chemical proceedings. An analytical approach to examine the character of water through development of a model describing the substantiality evolution was exhibited^17^. Taner et al.^34^ assessed the quality of water through a determinstic tool reinforcing the decision process. An approach of an estimation of minimum root mean error square through Artificial Neural Network^35^ was used to assess the quality of water in a data-scarce region. However, the complexity of establishing a theoretical model illustrating the course of progression through multiple stages has redirected the inquiry to appraise the water quality through data interpretation. Moreover, readjustment of model parameters in accordance with geographical, chemical and biological alterations of parametric values required considerable amount of time and strenuous mathematical build-up. A shift towards the data estimation of water stature through multi-parametric assessment approach, contrasting the univariate approach^13^ was observed. The potentiality of statistical methods to analyze a monumental multi-parameters data with error precision was employed^1,2,5,11,23,29,36^ in the proceedings of water-data valuation^19^. used a combination of factor analysis and spatial distribution of water parameters to assess the quality of some locations of Aurangabad district in India. It was observed by^39^ that major contamination threat to Karoon River was caused by geological situation for over the year which was defined as nonpoint pollution source and explained the most part of the observed variances (50%) in the data. The analysis and results were obtained using factor analysis^38^. integrated factor analysis method in fuzzy comprehensive evaluation and orthogonal experimental design to calculate water breakthrough time, stable production period and gas recovery for different cases under different parameter combinations. This analysis supplied theoretical basis for preventing and controlling the water invasion thus improving the development effect of condensate gas reservoir with bottom water. An infusion of networking modelling and Spearman correlations^15^ was adopted to assess the influence of parameters on water quality.

Recently, many scholarly efforts have been made to assess the quality of water using machine learning and artificial intelligence schemes^9^. proposed that the classification of self-organizing map (SOM) could express the comprehensive water quality in a water distribution network (WDN) directly and vividly by high-dimension water quality indicator projection to a low dimensional topology grid. It was further established that two-stage classification using SOM and clustering methods provided higher efficiency in comparison to the traditional clustering method^40^. reviewed the cases in which machine learning algorithms were applied to evaluate the water quality in different water environments, such as surface water, groundwater, drinking water, sewage, and seawater. Three machine learning models including boosted regression trees (BRT), multivariate discriminant analysis (MDA), and support vector machine (SVM) were used by^26^ to develop a novel framework for risk assessment of nitrate groundwater contamination. It was established that the accuracy for the three models ranged from 0.81 to 0.87, therefore all models were considered for ensemble modeling process. It was maintained in^31^ that Data-driven modelling enabled water producers to interpret the measurements in the context of what concentrations could be expected based on the recent historic data, and thus identified unexplained deviations warranting further investigation of their origin. An applicability of ML algorithms to asses medical, agricultural finance and sports datas’ trend and to avail the models predictability was discussed by^6–8^. A new taxonomy of ML algorithms was provided in^24^ to highlight the importance of fine algorithm development to overcome the challenges like topology changes, link failures, memory constraints, interoperability, network congestion, coverage, scalability, network management, security, and privacy. In view of these computing explorations to assess data parameters’ correlation and their propensity to influence the system, we intend to use a combination of statistical inference and ML algorithms to study an aquatic ensemble. An investigation of creating a linking mechanism between unsupervised machine learning and statistical analysis approach is conducted in order to seek freedom of comparable use of either of the schemes/methods relative to data feasibility. A higher performance accuracy of an ML method and its higher comparability probability with multivariate statistical approach will be required to generate state of the art (SOTA) analysis. The mechanism of ML methods to learn the structure of unlabelled observations in a data facilitates the computation of predictive accuracy of results through receiver operating curve (ROC) and support vector machine (SVM), henceforth providing the basis for SOTA precision.

The dual validating approach of assessing and interpreting a data using collusion of ML and (statistical analysis) SA schemes has been introduced here and implemented on water concentration data. However, the urgency and demand of conclusive results for a sensitive data necessitates the higher performance precision and predictive accuracy. A computing efficient approach highlighting anomalous contents of the data can be used in medical sciences for tumor detection, for assessment of febrile survival period in early stages of Acute Lymphoblastic Leukemia and for image analysis in web security tasks.

The current article presents a consolidation of statistical disposition and machine learning (ML) tools to attain an impertinent understanding of a raw data. Water components’ data for 16 strategically chosen locations of Lahore, the capital of second most populated province of Pakistan, has been considered for experimentation. The objectives of the study are

- An analysis of the data will be performed to accomplish a twofold objective of assessing the water quality and illustrating the discerning ML schemes, aided by multivariable statistical inference.
- The propriety and suitability of each computational method will be examined prior to its application on the data.
- In view of the probabilistic inferences made by statistical methods and their continuation in ML decision-process, a spectrum of colluding results will be built to further the process of water valuation. Each method’s quantitative and descriptive results will be ruminated to highlight the significant features of the data.
- A feature of redundant variables’ renouncement will be incorporated in the analysis through identification of clustering movement of similar characteristic variables.
- A range of data visualization methods will be adapted to display the results obtained by fusion of ML and statistical methods.
- An innovative approach of mutually validating ML and SA (statistical analysis) schemes will be presented in order to prepare the groundwork for (state of the art) SOTA analysis. The advantage of our approach can be examined through the fact that related SOTA analysis will further refine the predictive precision between two comparable methods, unlike the SOTA between two random ML methods.

## Materials and methods

**Figure 1:**
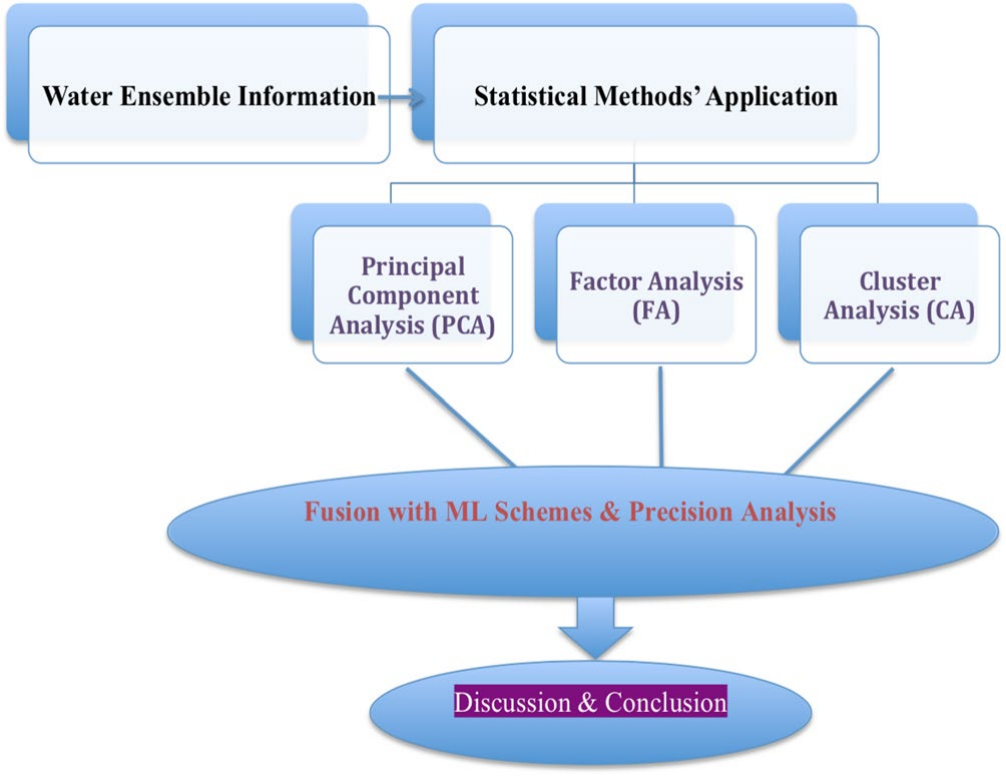
Study flow chart.

A study flow chart, Fig. 1, has been displayed below

### Study zone

The drinking water quality of the captial of province Punjab, Lahore, the second largest north-eastern city (lying between $31^{\circ }15^{'} - 31^{\circ }45^{'} N$ and $74^{\circ } 01^{'}-74^{\circ }39^{'} E$) of Pakistan, has been monitored and assessed. It is one of Pakistan’s most socially liberal, progressive and cosmopolitan cities with the area of 684 ${\text {miles}} ^{2}$. An assessment of drinking water of the city is conducted to make a contribution to a nationwide water quality-monitoring program initiated in 2002 by Pakistan Council of Research in Water Resources (PCRWR) focusing on 24 major cities of the country. The outcomes of the program, shared through five-years assessment reports, help raise awareness in the country and act as a guideline for the federal, provincial and local governments to take necessary steps towards improvement in the water quality. Moreover, the rationale of current study of samples from 16 strategic locations of the city is to assess the change in water quality decline, observed in the years 2003-2006^16^. It was noted that excess of Arsenic, Turbidity, Iron, TDS and bacteria in 31% of these locations rendered them a status of compromised water quality stations. A cumulative set of efforts and water treatment procedures improved the quality of water in these locations by a margin of 20% by the end of 2018. However, an assessment of aqua quality conducted in 2018 demonstrated a slight quality decline in five of these sixteen locations. The current analysis is prompted to determine a categorical assessment of improved quality in the marked locations after concerted efforts of water treatment procedures.

### Monitoring sites

In pursuit of the program National Level Water Quality Monitoring 2020, Pakistan Council of Research in Water Resources (PCRWR) had set up a monitoring station in Regional Office Lahore to collect water samples from strategically selected sites of the city. To collect water samples, a grid size of 16 $km^2$ was chosen and 16 sampling locations were elected based on their representative nature of water source, treatment plant, storage facilities, network distribution and consumer delivery & use points.

Adopting a uniform methodology in accordance with APHA (American Public Health Association) 2017 protocols, four types of samples were collected from each site. The classification of these types^16^, aiming to distinguish between different purpose testing, is given in Table 1.

**Table 1:** Classification of sample types.

Types	Purpose of testing
A	Microbiological testing
B	Trace elements
C	Nitrate
D	Physio-chemical parameters

Prior to collecting samples and adding preservatives for types B, C & D testing, proper care was administered to have the bottles thoroughly rinsed with deionized water. The transportation of samples collected for chemical analysis was conducted without iceboxes. To prevent the color from changing during storage and transportation, residual chlorine, pH and turbidity were tested immediately after sample collection. A controlled temperature of 2 °C and 8 °C was maintained while transporting the type A samples in disinfected and insulated boxes of meticulous lightproof environment. Following APHA 2017 protocols, the time between sample collection and its analysis did not exceed beyond 6 hours. Water samples from the tube wells of 16 significant tabbed locations in the city were collected, and for factual ground water representation it was allowed to flow for at least 10 min before the sample collection.

### Data constituents

**Figure 2:**
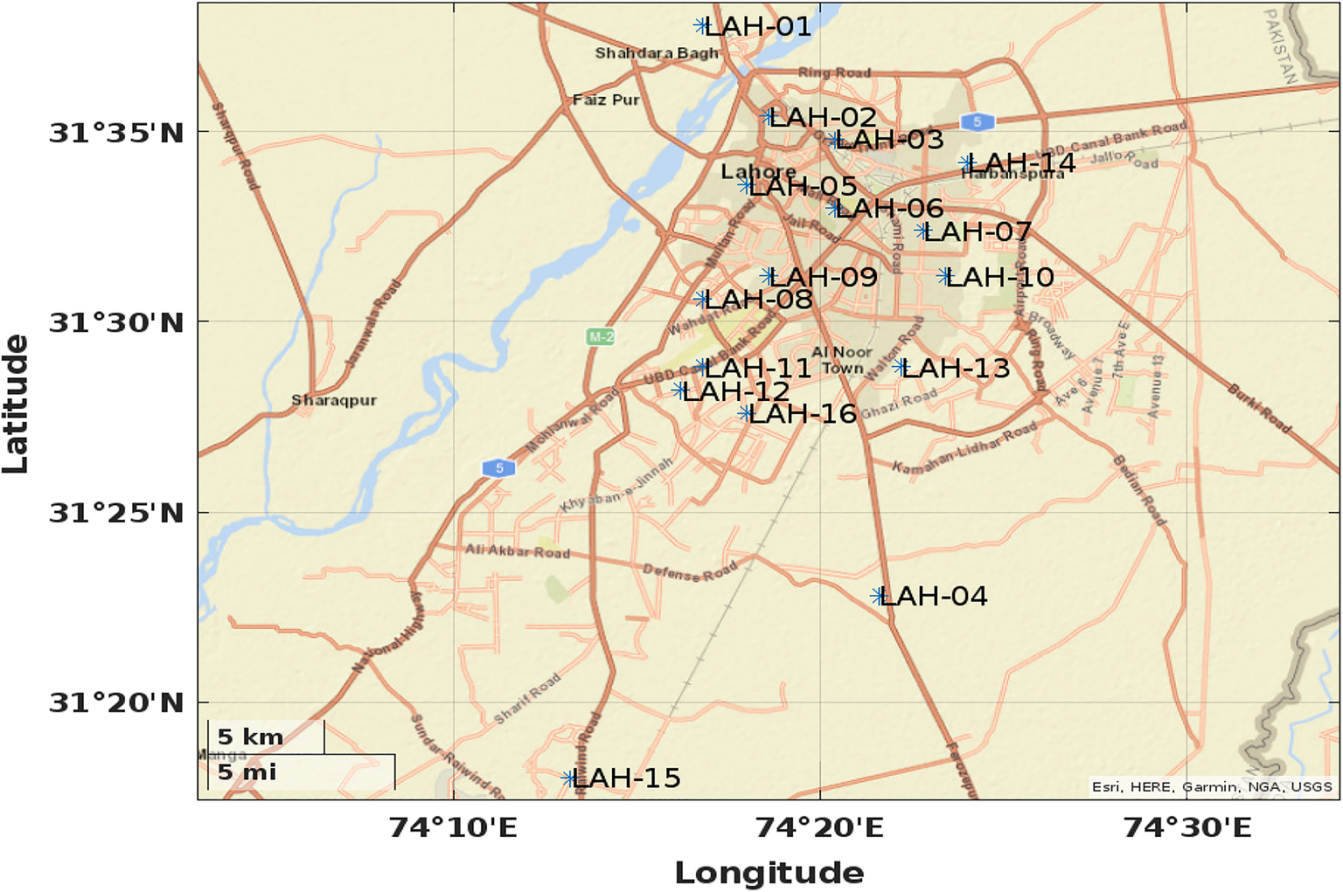
Coordinates placement of electic locations in Lahore (https://www.mathworks.com/help/matlab/ref/geobasemap.html).

The current data relates to the water samples collected in year 2020^16^ by PCRWR from 16 specific locations in the city of Lahore. These locations have been name coded in Table 2. and their coordinates placement in the map has been shown in a map^22^, Fig. 2. To appraise the quality of collected water samples, the parametric values of elements Electrical Conductivity (EC), pH, Turbidity, Bicarbonate ($HCO_3$), Carbonate ($CO_3$), Calcium (Ca), Magnesium (Mg), Hardness (Hard), Chloride (Cl), Sodium (Na), Potassium (K), Sulphate ($SO_4$), Nitrate ($NO_3$), TDS, Iron (Fe), Fluoride (F), Arsenic (As), Total Coliforms and E. Coli have been assessed. Table 3 displays these parameters along with the measuring units and diagnostic techniques utilized to gauge their concentration in the water.

### Statistical methods’ application and analysis

To evaluate the water parameters data for the twofold purpose of highlighting its features and determining its quality in comparison to the international standards, a few statistical methods have been employed. A spectrum of resulting attributes from each of these methods shall be built to establish the bordering water stature in the city. The use of numerical methods and their inferences have been detailed subsequently.

**Table 2:** Collected samples’ locations in Lahore^16^.

Location code	Location name
LAH-01	Old Shahdara Town Centre Tubewell
LAH-02	Ali Park Tubewell-1 Fort Road
LAH-03	Sultanpura Tubewell Near Chah Meeran Shah
LAH-04	Goharabad Tubewell Shalimar Town
LAH-05	Tubewell Riwaz Garden
LAH-06	Tubewell Federal Lodge Chamba House
LAH-07	Tubewell Tufail Road Saddar Bazar
LAH-08	Tubewell-12 Ravi Block Allama Iqbal Town
LAH-09	Goal Bagh Tubewell Wahdat Colony
LAH-10	Guromanget Tubewell Gulberg-III
LAH-11	PCSIR Housing Society Canal Bank Road
LAH-12	LDA Flats opposite Faisal Town Ghosia Masjid
LAH-13	Farooq Colony Walton Road Police Line
LAH-14	Tubewell Cantonment Road Asghari Flats
LAH-15	Punjab Govt. Cooperative Housing Society
LAH-16	Govt. Housing Scheme Township A-1

**Principal component analysis (PCA)**

In PCA^37^, an original set of variables is transformed into a new set of variables, called principal components. Each principal component is the linear combination of original variables. These principal components represent an orthogonal basis for the whole dataset.

**Figure 3:**
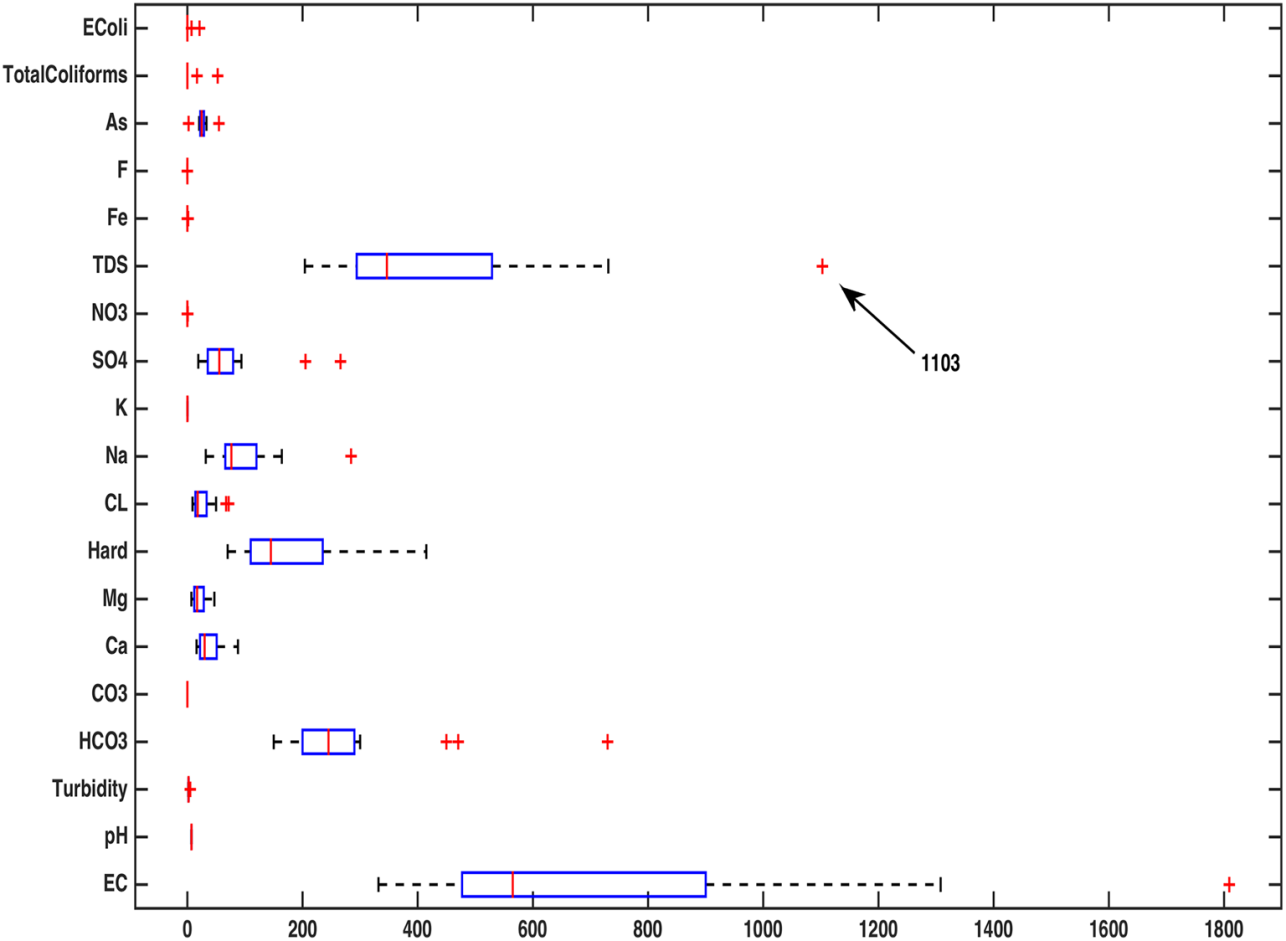
A boxplot showing water parameters’ variability.

A boxplot of our data has been obtained to reflect on some significant information about water quality variables. Figure 3 indicates that the parameters pH, Turbidity, $CO_3$, K, $NO_3$, Fe, F, As, Total Coliforms and E. Coli could be forfeited on account of their constancy at several locations. A higher variability in EC and TDS occupation in water samples has been observed as compared to the elements of $HCO_3$ and Hard. This figure also points out an outlier for TDS at 1103 on location LAH-13 that has been found to exceed the maximum permissible limit 1000mg/l NDWQS (National Drinking Water Quality Standards of Pakistan) for healthy drinking water.

**Figure 4:**
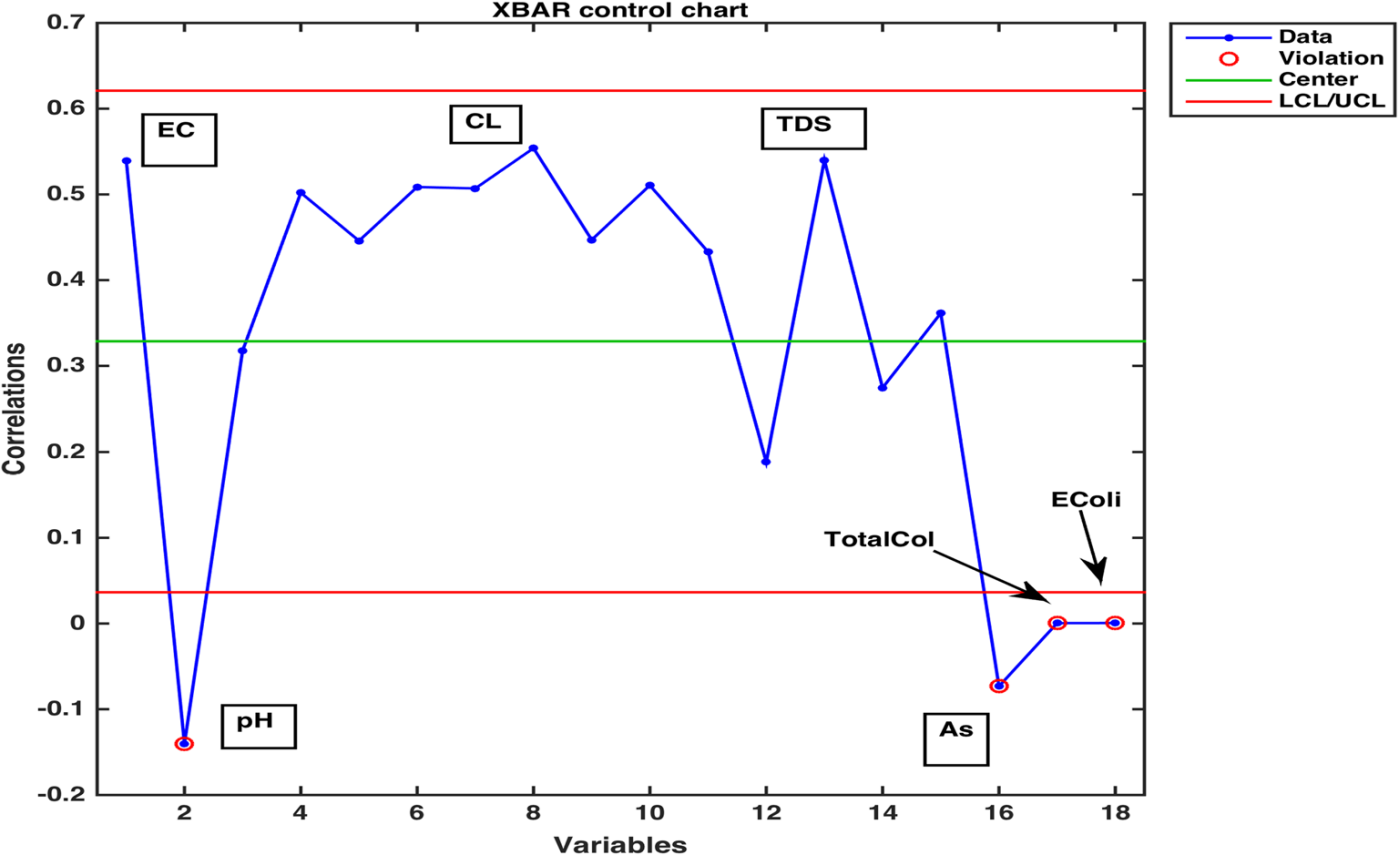
A display of peaked and based correlations.

**Figure 5:**
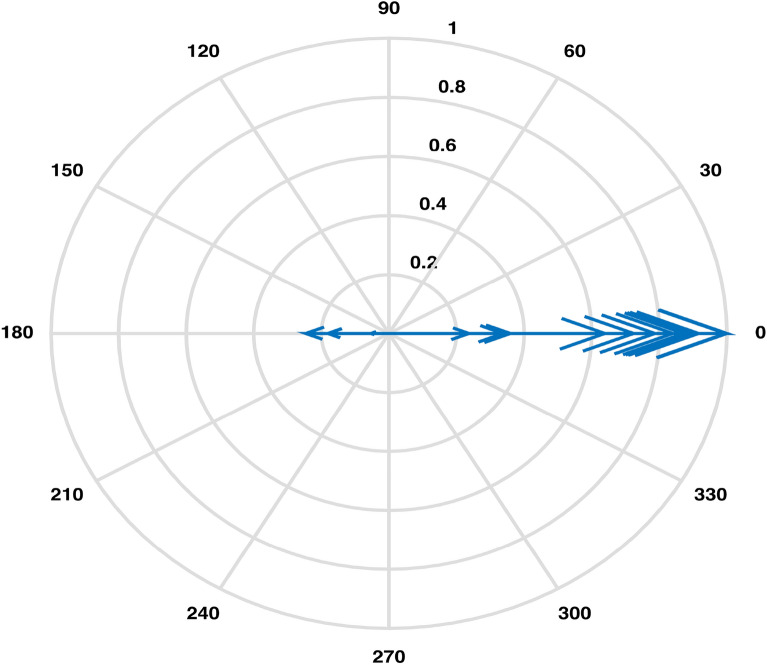
Pairwise correlations of EC with other variables.

**Figure 6:**
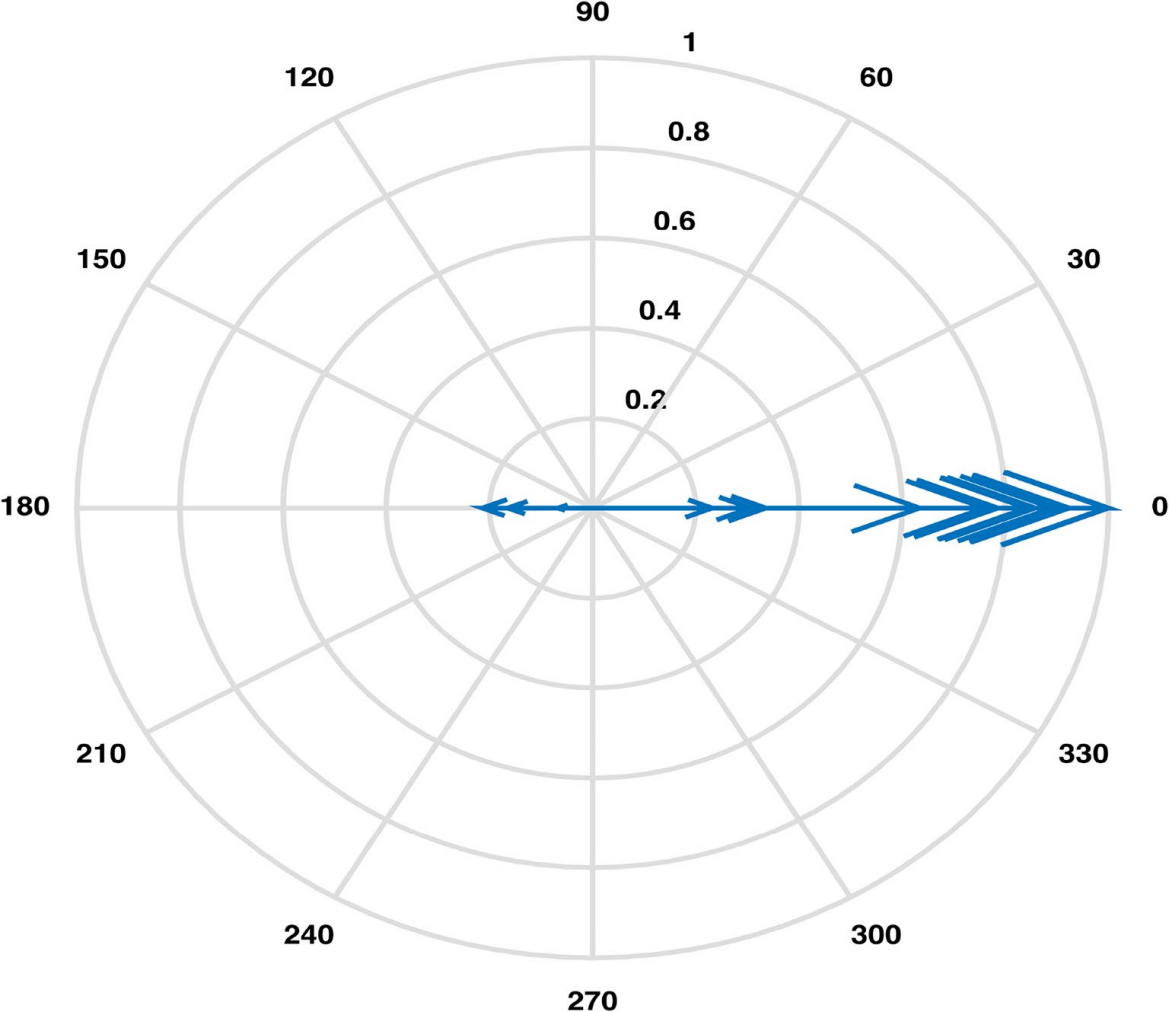
Pairwise correlations of TDS with other variables.

**Figure 7:**
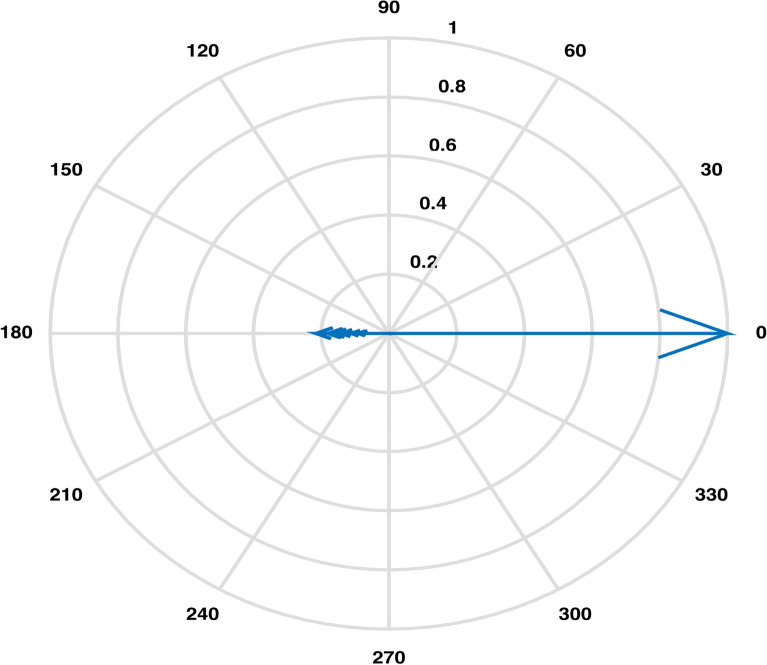
Pairwise correlations of total coliforms with other variables.

**Figure 8:**
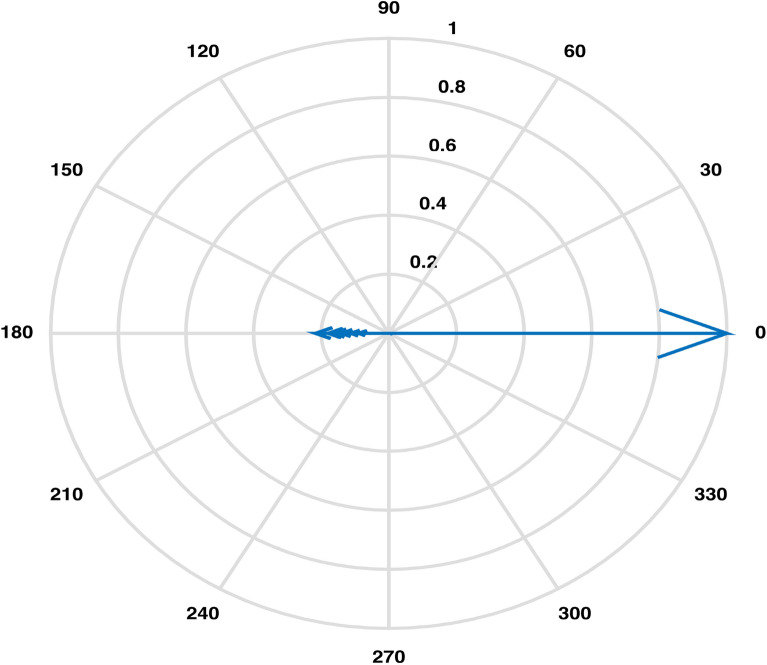
Pairwise correlations of E. Coli with other variables.

An estimation of pairwise correlations between variables in the data (except for $CO_3$) has been obtained. It is observed that some variables have higher correlation such as 0.99 between EC and TDS, as compared to some other variables. An XBAR control chart Fig. 4 of these correlations illustrates the highly correlated variables EC, Cl and TDS while pH, As, Total Coliforms and E. Coli are shown to be the least correlated variables due to having fallen in the category of below zero correlations. Higher pairwise correlations of EC and TDS with other variables can also be observed through their compass diagrams Figs. 5 and 6 where 61% of the values are contained in between the circular strips of radii 0.5 and 1. Also, lowest pairwise correlations of Total Coliforms and E. Coli with other variables can be observed through their compass diagrams Figs. 7 and 8 where concentration on $180^{\circ }$ indicates the negative correlations.

**Figure 9:**
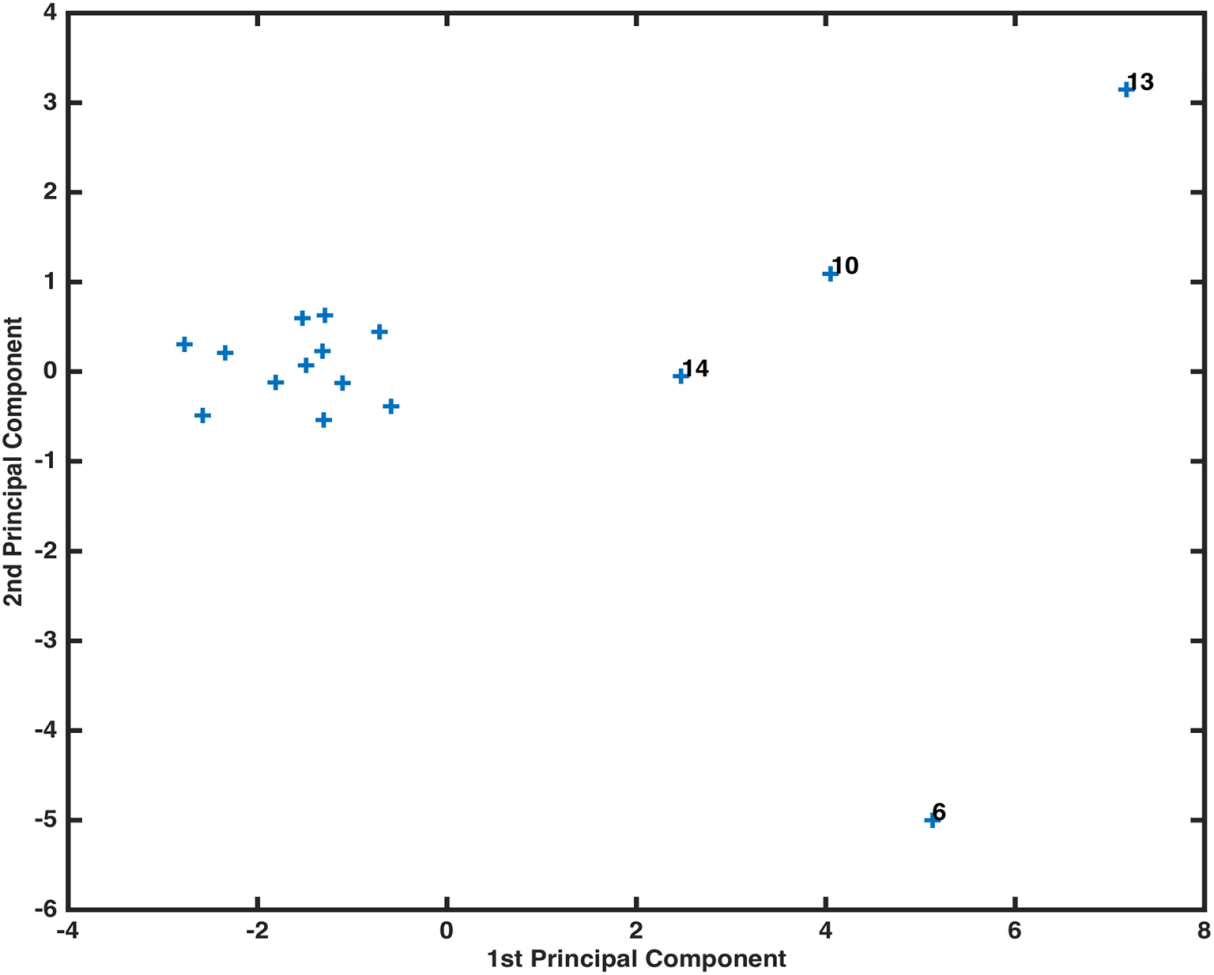
Components plot.

We performed the principal component analysis by using the inverse variances of water components’ values as weights. The five outputs of PCA are subsequently described. The first output encompasses weighted coefficients of principal components of original variables. These coefficients are transformed to form an orthonormal basis. The second output of PCA ‘component score’ describes the coordinates of the original data into new coordinate system defined by the principal components. The component plot Fig. 9 shows centered and scaled data projected onto the first two principal components. It can be observed that the water samples collected from locations LAH-06, LAH-10, LAH-13 and LAH-14 show extreme tendency as compared to other locations’ samples.

The third output of PCA ‘latent’ calculates the variances described by the corresponding principal components. Here for each column of score, there is a sample variance equal to the corresponding row of latent. In order to measure the multivariate distance of each observation from the center of the data, the fourth output of PCA, tsquared or Hotelling’s $T^2$, has been calculated. This is also an analytical approach to calculate the extreme points of the data. The fifth and final output of PCA ‘explained’ contains the percent variances described by the corresponding principal components.

**Table 3:** Water components, their measuring units and diagnostic procedures.

Parameters	Abbreviation	Units	Diagnostic techniques
Electrical conductivity	EC	$\mu$S/cm	EC Meter Hach-44600-00 USA
pH	pH	pH unit	pH Meter Hanna Instrument, Model 8519 Italy
Turbidity	Turbidity	NTU	Turbidity Meter Lamotte, Model 2008 USA
Bicarbonate	$HCO_3$	mg/l	2320 Standard Method APHA 2017
Carbonate	$CO_3$	mg/l	2320 Standard Method APHA 2017
Calcium	Ca	mg/l	3500-Ca-D Standard Method APHA 2017
Magnesium	Mg	mg/l	2340-C Standard Method APHA 2017
Hardness	Hard	mg/l	EDTA Titration Standard Method APHA 2017
Chloride	Cl	mg/l	Titration Standard Method APHA 2017
Sodium	Na	mg/l	Flame Photometer PFP7 UK
Potassium	K	mg/l	Flame Photometer PFP7 UK
Sulphate	$SO_4$	mg/l	SulfaVer4 Hach-8051 by Spectrophotometer
Nitrate	$NO_3$	mg/l	Cd. Reduction Hach-8171 by Spectrophotometer
TDS	TDS	mg/l	2540C Standard Method APHA 2017
Iron	Fe	mg/l	Spectrophotometer Standard Method APHA 2017
Fluoride	F	mg/l	4500-FC Ion-Selective Electrode Standard Method APHA 2017
Arsenic	As	$\mu$g/l	AAS Vario 6, Analytik Jena AG 3111B APHA 2017
Total coliforms	TotalColiforms	CFU/100ml	9221-B, C & D, Standard Method APHA 2017
E. Coli	EColi	CFU/100ml	9221-B, C & D, Standard Method APHA 2017

**Figure 10:**
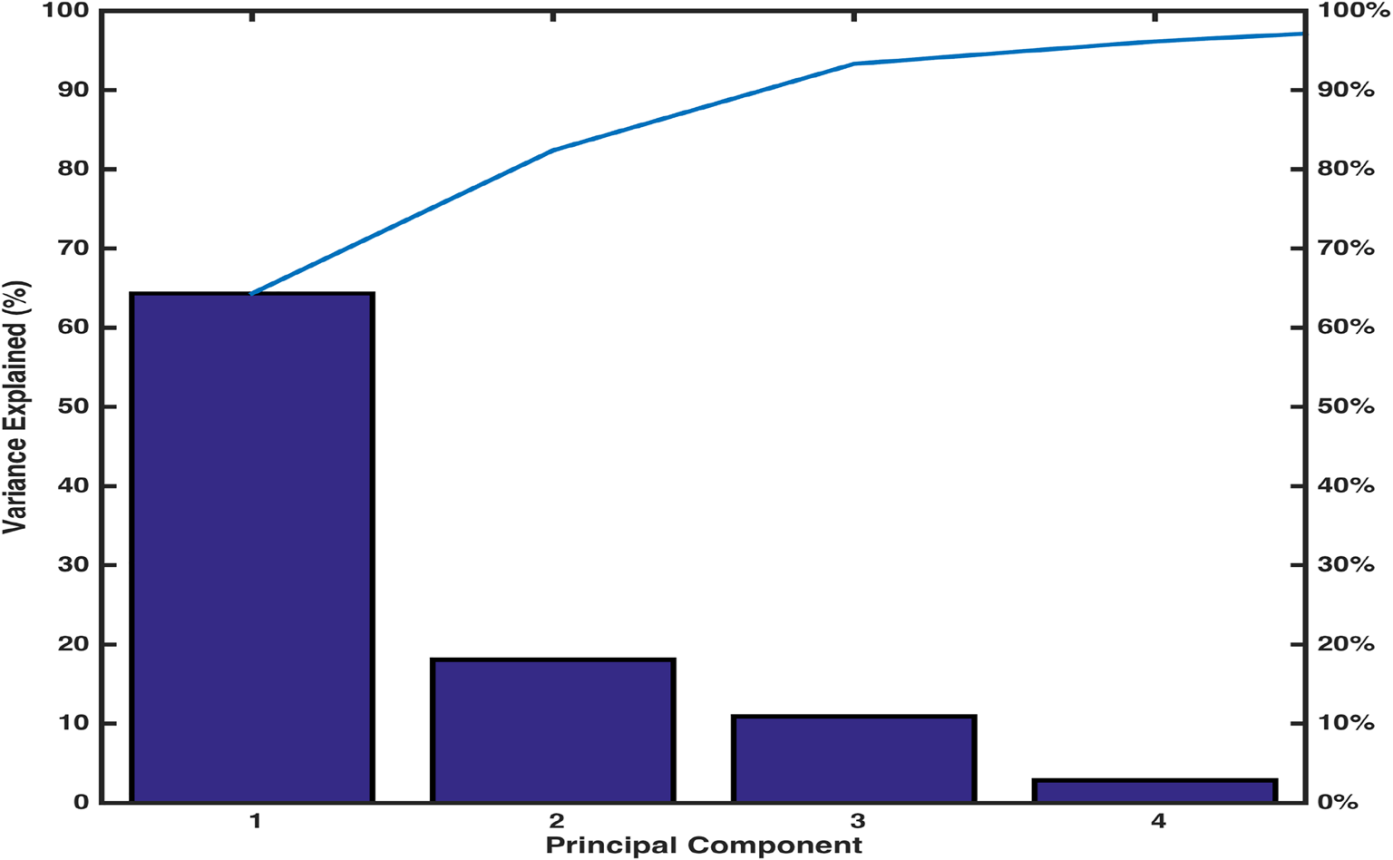
Scree plot.

**Figure 11:**
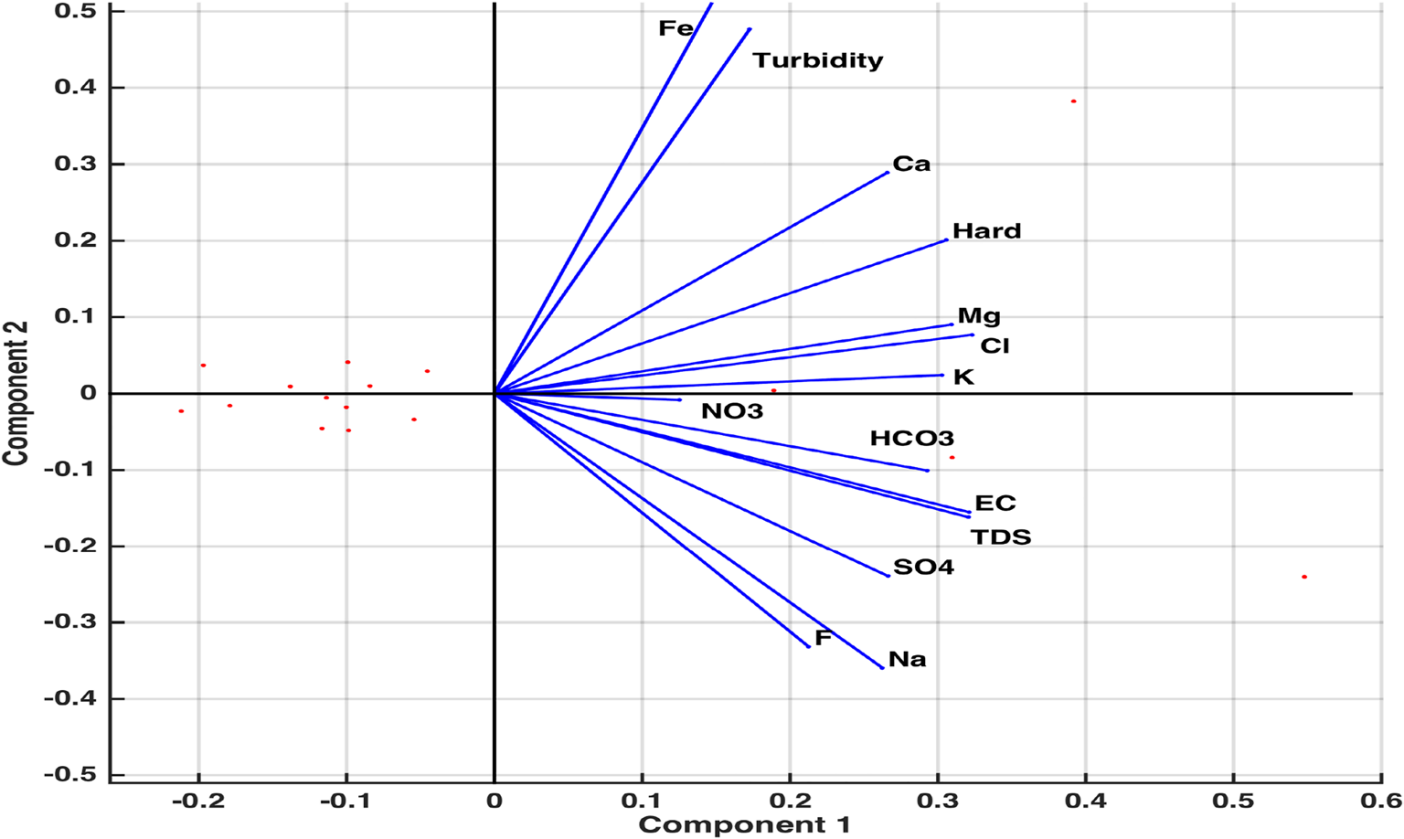
Principal component coefficients & principal component scores plot.

A Scree plot Fig. 10 representing the explained variance corresponding to each principal component displays that 95% of the total variance is explained by first four principal components. The first principal component is accounting for 62% of the variance, postulating the need for more components. Based on the numerical figures obtained through PCA, a bi-plot Fig. 11 exhibiting the orthonormal principal component coefficients for each variable and the principal component scores for each observation simultaneously is laid out. It is observed that the variables EC, TDS, Mg and Cl contribute highly to the first principal component having highest coefficient values. Also, it can be seen that two sets of variables are distinguished by second principal component such that Fe, Turbidity Ca, Hard, Mg, Cl, K are defined by the positive coefficients & $NO_3$, $HCO_3$, EC, TDS, $SO_4$, Na and F are defined by the negative coefficients.

**Factor analysis (FA)**

Factor analysis^10,32^ is applied to a multivariate data to gauge the interdependence of a group of variables on some other variables. In this test, the measured variables depend on a smaller number of factors known as common factors. The main outputs of this analysis are loadings (Table 4) and specific variance (Table 5); loadings are the coefficients when each measured variable is expressed as a linear combination of common factors and specific variance is a component associated with each measured variable characterizing its independent variability.

For factor analysis, the variables having specific variance less than 0.005 have been forfeited and the remaining variables’ significance in predicting the overall quality of the data has been discussed. A machine-learning program factoran has been used to calculate the outputs of loadings, specific variance, T-stats and factor scores. It can be observed through the figures of specific variance that except for the variable *Mg*, all other variables are largely determined by five common factors. The corresponding value $p=0.1916$ also suggests the failure of null hypothesis of 5 common factors, implying this model’s potential to make accurate prediction of the water quality data.

**Table 4:** Loadings matrix for factor analysis.

Variables	Factor 1	Factor 2	Factor 3	Factor 4	Factor 5
EC	0.94	0.07	−0.30	−0.05	−0.09
Turbidity	0.41	0.83	0.34	−0.17	0.04
Ca	0.70	0.50	0.14	0.47	−0.07
Mg	0.91	0.20	0.06	0.06	0.019
Cl	0.91	0.31	−0.09	0.20	0.06
K	0.88	0.17	−0.02	0.02	−0.34
$SO_4$	0.92	−0.35	−0.07	−0.14	−0.00
$NO_3$	0.61	−0.53	0.57	0.06	0.06
Fe	0.36	0.76	0.44	0.01	0.18
F	0.54	0.05	−0.79	0.08	0.26

**Table 5:** Specific variance for factor analysis.

Variable	EC	Turbidity	Ca	Mg	Cl	K	$SO_4$	$NO_3$	Fe	F
Specific variance	0.005	0.005	0.013	0.132	0.018	0.074	0.005	0.005	0.058	0.013

**Table 6:** Loadings matrix in rotated coordinates system.

Variables	Variance F1	Variance F2	Variance F3	Variance F4	Variance F5
EC	**0.74**	0.01	0.35	−0.03	−0.042
Turbidity	0.19	**1.06**	−0.12	−0.18	−0.14
Ca	0.18	0.05	−0.01	−0.00	**0.84**
Mg	**0.34**	0.27	0.20	0.25	0.13
Cl	0.21	0.19	**0.41**	0.11	0.34
K	**1.13**	-0.06	−0.23	-0.13	0.17
SO_4_	0.49	−0.05	0.28	**0.52**	−0.25
NO_3_	−0.10	−0.08	−0.24	**1.11**	0.06
Fe	−0.27	**0.99**	−-0.01	0.08	0.09
F	−0.11	−0.12	**1.137**	−0.21	0.00

The variables EC, Mg and K are in the column Variance F1, and they display the highest magnitude in their respective rows in comparison to Variance F2, Variance F3, Variance F4 and Variance F5. These values are highlighted to indicate that EC, Mg and K are explained by 1st factor. Similarly, Turbidity and Fe are explained by 2nd factor (in column Variance F2); Cl and F are explained by 3rd factor (in column Variance F3); SO_4_ and NO_3_ are explained by 4th factor (in column Variance F4), and Ca is explained by 5th factor (in column Variance F5).

**Figure 12:**
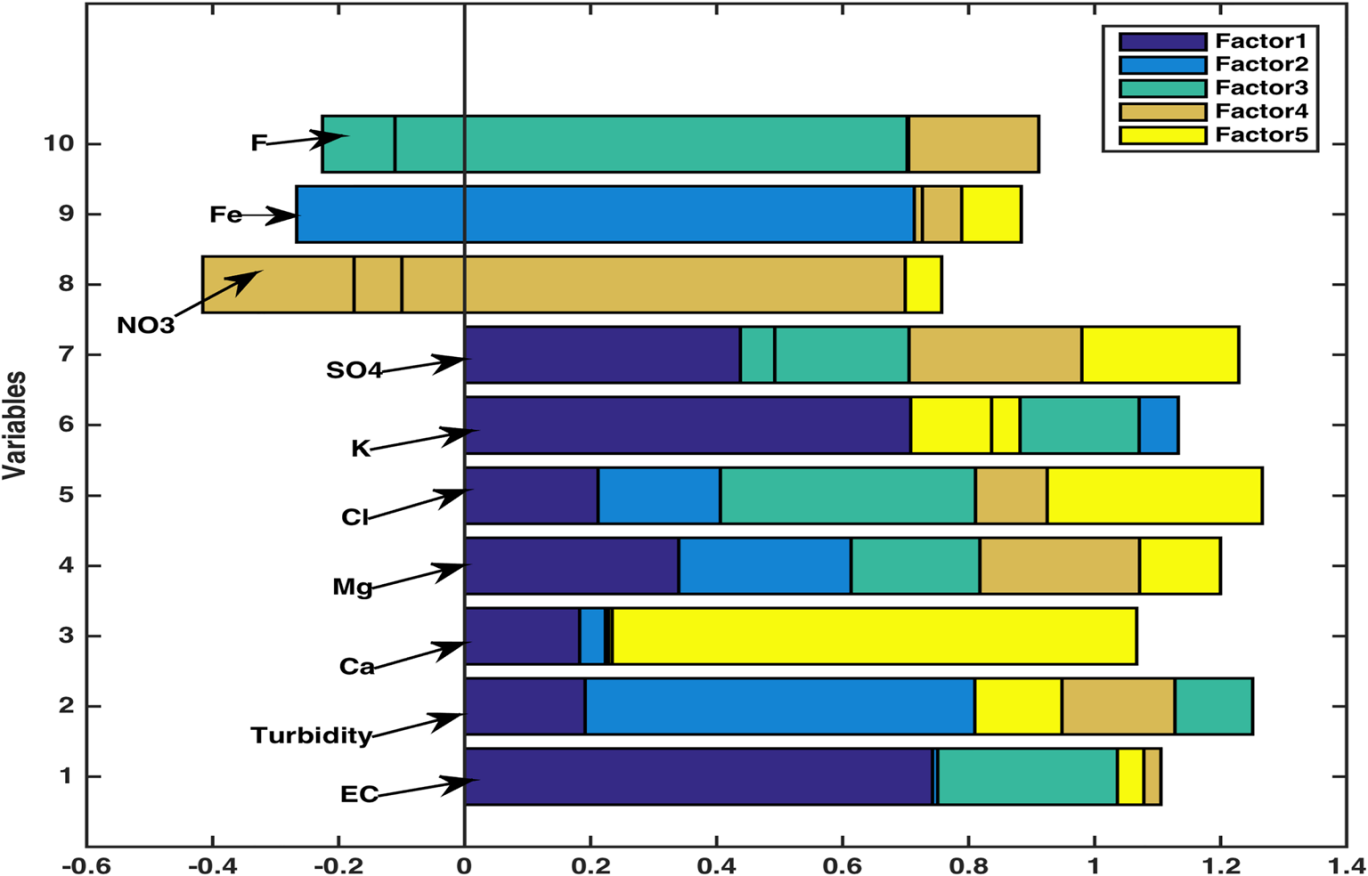
Rotated estimates of loadings.

In order to obtain the loadings in rotated coordinate system (Table 6) such that each variable is affected by only one factor, we use promax criterion, a common oblique method. Fig. 12 of rotated estimates of loadings has been obtained to show that the variables EC, Mg and K are explained by 1st factor; Turbidity and Fe are explained by 2nd factor; Cl and F are explained by 3rd factor; $SO_4$ and $NO_3$ are explained by 4th factor, and Ca is explained by 5th factor. Here first, second, third, fourth and fifth factors are identified with purple, blue, green, brown and yellow colors, respectively.

**Figure 13:**
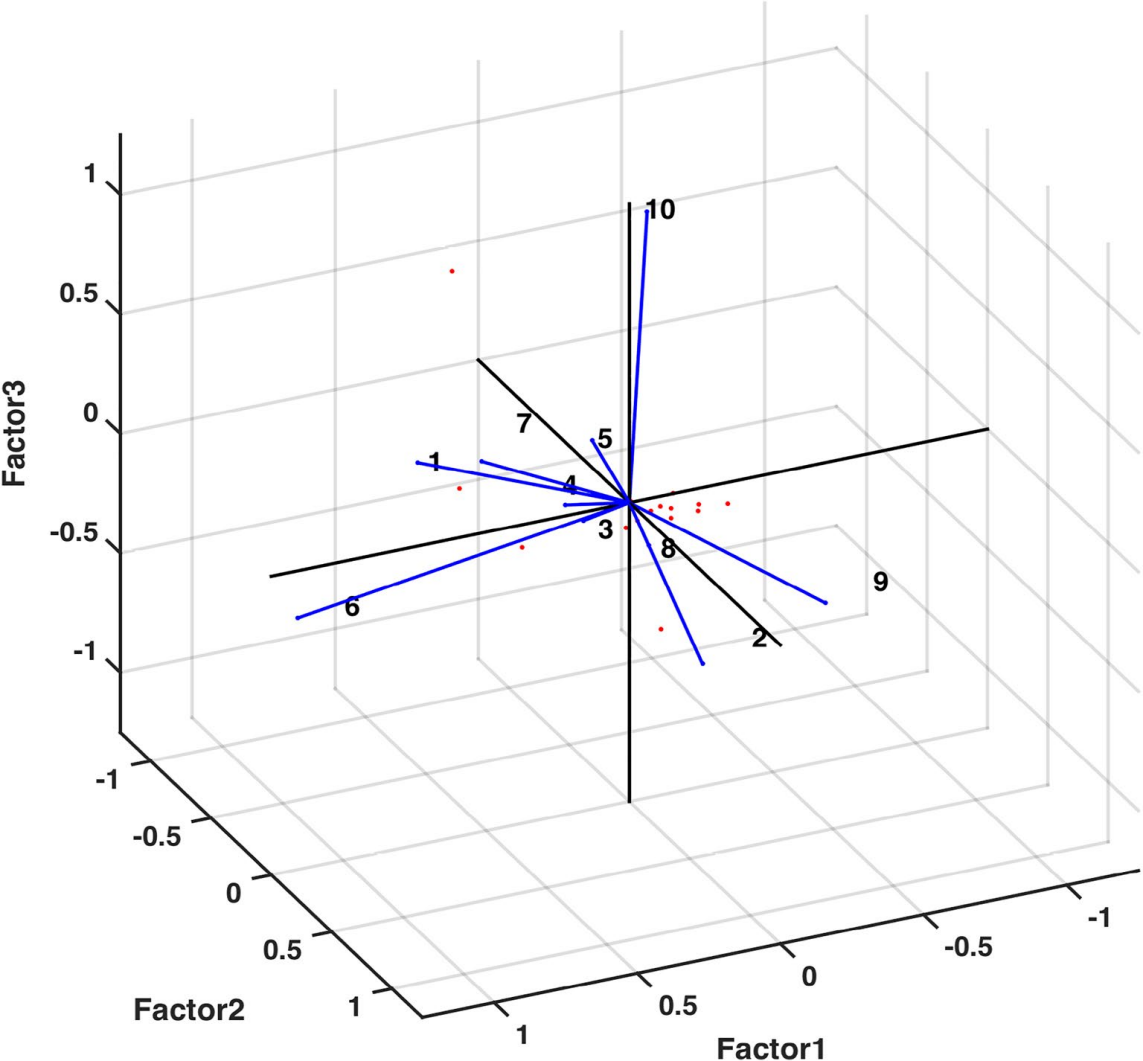
Rotated factors (1–3) score plot.

**Figure 14:**
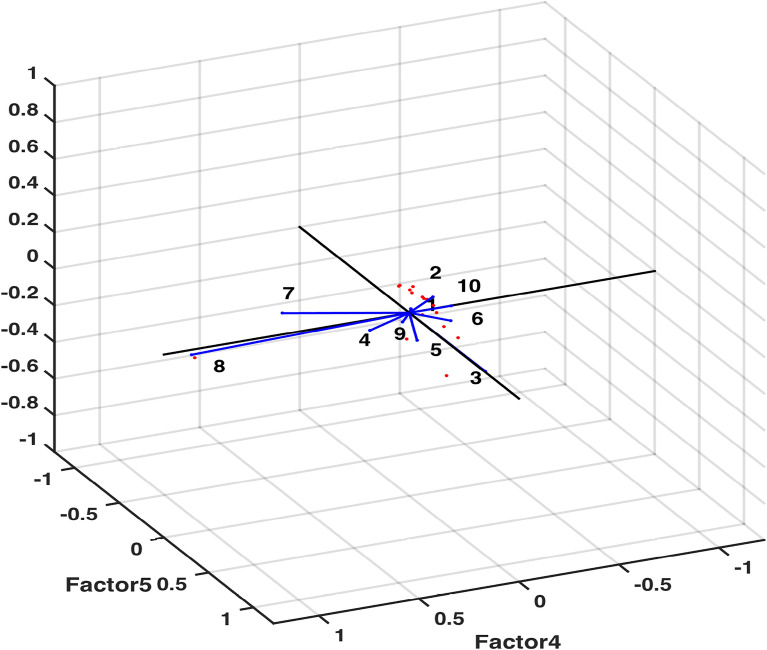
Rotated factors (4–5) score plot.

Also in order to assert the factors affecting the variables, rotated factor scores have been obtained and used to plot against corresponding factor loadings to further analyze the data. Through Fig. 13, we have sought the variables affected by first three factors only. It can be observed that the variables EC (1), Mg (4) and K (6) are influenced by Factor 1, the variables Turbidity (2) and Fe (9) are influenced by Factor 2, and the variables Cl (5) and F (10) are influenced by Factor 3. Fig. 14 shows that SO_4_ (7) and NO_3_ (8) are influenced by Factor 4, and the variable Ca (3) is influenced by Factor 5.

**Cluster analysis (CA)**

To investigate the similarity of variables such that the influence of a cluster of variables sharing similar characteristics on the whole model could be examined, we performed hierarchical clustering^21,30^ on water components data. This analysis was conducted on the variables in Table 7.

**Table 7:** Numbers assigned to variables for cluster analysis.

Variable	EC	pH	Turbidity	$HCO_3$	Ca	Mg	Hard	Cl	Na	K	$SO_4$	$NO_3$	TDS	Fe	F	As	TotalColi	EColi
Assigned number	1	2	3	4	5	6	7	8	9	10	11	12	13	14	15	16	17	18

In hierarchical clustering, grouping of the data is performed over a variety of scales by constituting a cluster tree. This tree is constructed such that the clusters at one level are joined with the clusters at another level, distinguishing between similar characteristics’ variables that influence the data as a group. A machine-learning program clusterdata has been used to prepare this analysis. Three main outputs of CA; pdist, linkage and dendrogram, demonstrating the distance between every two pairs of variables, linking of close proximity variables and clustering tree respectively, were attained.

To conduct agglomerative cluster analysis on our data, we normalized the data values as different variables were scaled differently due to their measuring units. A dissimilarity matrix was obtained providing the distance between every two pairs of variables in the data set of 18 variables. A linkage function has been used to create binary clusters employing a pair of variables, and then linking these clusters with larger clusters such that a hierarchical tree of variables’ grouping is constructed.

The linkage function caters to Table 8 where each row identifies a link between two variables or clusters. If two variables are grouped by a link then a unique cluster index is assigned to this pair to continue linking this cluster to the other clusters/variables. The third column of this table shows the distance between two variables or clusters. The first value in the third column presents the closest proximity distance between two variables in the data such as $NO_3$ and F.

**Table 8:** Distance between two variables/clusters.

Variable 1	Variable 2	Distance
12 ($NO_3$)	15 (F)	0.00
10 (K)	19	0.00
14 (Fe)	20	0.00
3 (Turbidity)	21	0.05
2 (pH)	22	0.14
18 (E. Coli)	23	0.17
6 (Mg)	8 (Cl)	0.19
17 (total coliforms)	24	0.27
5 (Ca)	16 (As)	0.31
25	26	0.31
27	28	0.37
9 (Na)	11 ($SO_4$)	0.84
29	30	0.98
7 (Hard)	31	2.42
4 ($HCO_3$)	32	2.56
13 (TDS)	33	2.93
1 (EC)	34	5.97

**Figure 15:**
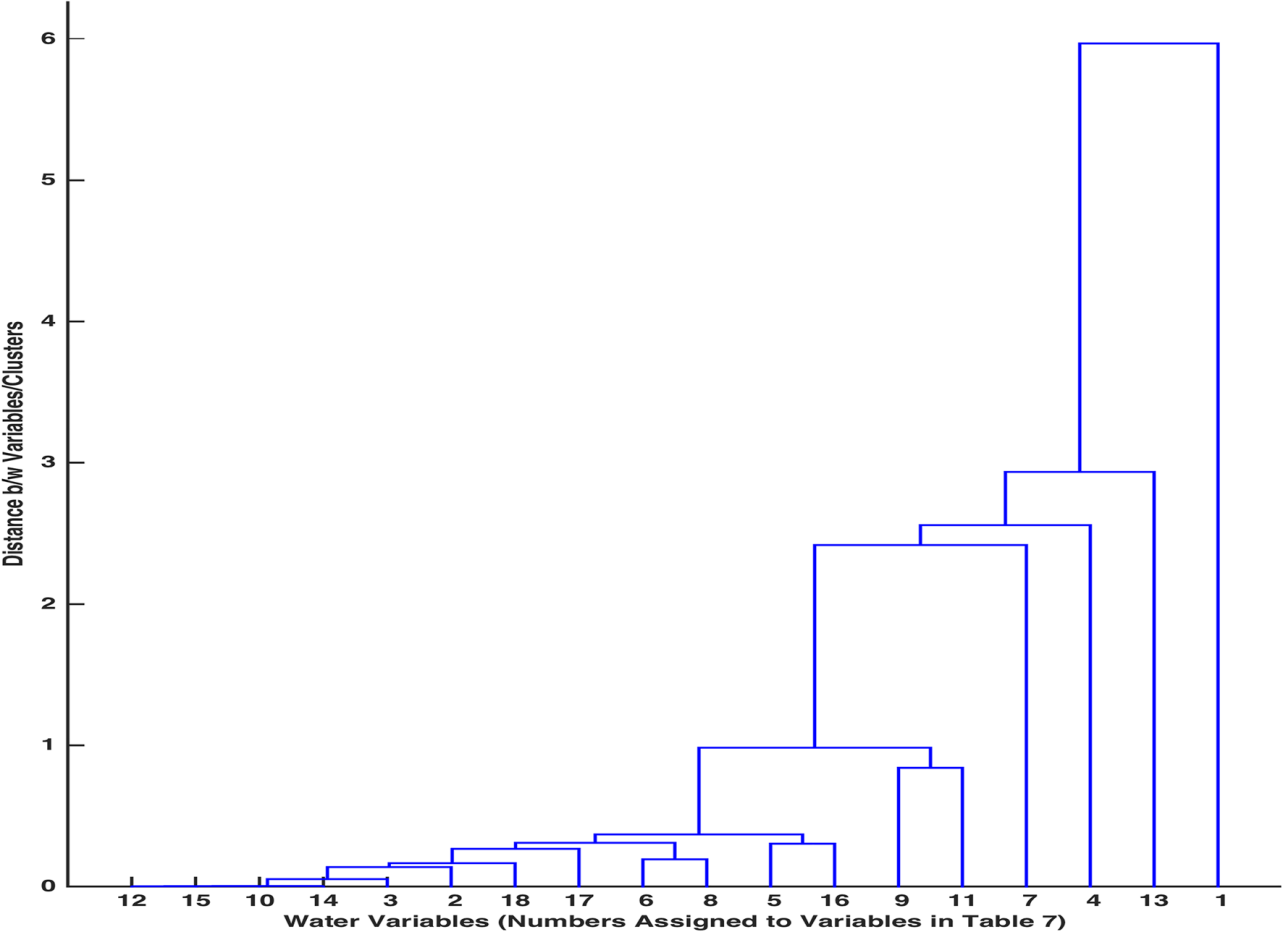
Dendrogram for clusters.

A hierarchical tree Fig. 15 involving the given set of variables has been displayed to reflect on the similarity of variables and an inconsistency of the links joining them. To verify that the clusters of variables created by the linkage function are suitable reflection of our data, a cophenetic correlation coefficient is calculated. This coefficient measures the correlation between distance matrix and linkage function matrix. For the obtained distance matrix and linkage matrix, the value of cophenetic correlation coefficient is $c=0.9582$ (closer to 1), indicating that the cluster tree (Fig. 15) authentically represents the actual variables’ distances.

An inconsistency measure is obtained to determine a natural division of clusters or to partition a dataset. This measure is mainly about comparing the height of a link with the height of other links below it. The smallest inconsistency measure between two variables (or two clusters) indicates that these two variables are highly indistinct belonging to a similar class or a cluster. For our data, an inconsistency function provides (18-1)$\times$ 4-matrix (Table 9 ) where 1st, 2nd, 3rd and 4th columns demonstrate mean of heights of links, standard deviation of heights of links, numbers of links used in the calculation and inconsistency coefficient, respectively. It can be observed from Fig. 15 that the given data variables $NO_3$ (12) & F (15), Mg (6) & Cl (8), Ca (5) & As (16) and Na (9) & $SO_4$ (11) are leaf nodes, having no variables below them with link inconsistency as zero. This feature is also verified by Table 9.

**Figure 16:**
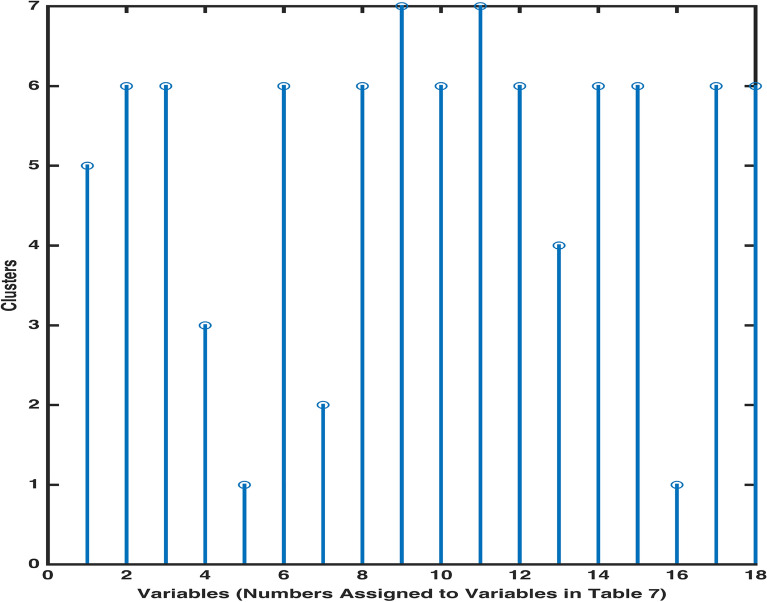
Stem diagram for clusters’ classification.

In order to determine the boundaries of clusters, where composing variables’ demonstrate different characteristics, we can employ inconsistency coefficient computation. Using an inconsistency coefficient 0.7842 as a cutoff for cluster boundaries, we observe that our given set of 18 variables can be placed in 7 clusters. A stem diagram Fig. 16 shows that the variables Na (9), $SO_4$ (11); pH (2), Turbidity (3), Mg (6), Cl (8), K (10), $NO_3$ (12), Fe (14), F (15), Total Coliforms (17), E. Coli (18); EC (1); TDS (13); $HCO_3$ (4); Hard (7); Ca (5), As (16) are placed in clusters 7, 6, 5, 4, 3, 2, 1, respectively.

## Water quality index

In order to classify water samples collected from 16 locations into grades I, II, III, IV as chronicled by^4^, we have computed water quality index (WQI) of each of these samples. The standard water grade division is described in Table 10.

**Table 9:** Inconsistency measure for the links in dendrogram.

Sr.	Mean of heights of links	SD of heights of links	Number of links	Inconsistency coefficient
1	0.00	0	1	0
2	0.00	0.00	2	0.71
3	0.00	0.00	2	0.71
4	0.03	0.03	2	0.71
5	0.10	0.06	2	0.71
6	0.15	0.02	2	0.71
7	0.19	0	1	0
8	0.22	0.07	2	0.71
9	0.31	0	1	0
10	0.26	0.06	3	0.89
11	0.33	0.04	3	1.15
12	0.84	0	1	0
13	0.73	0.32	3	0.78
14	1.70	1.02	2	0.71
15	2.49	0.10	2	0.71
16	2.75	0.26	2	0.71
17	4.45	2.15	2	0.71

**Table 10:** Standard water quality grade^4^.

Water quality index	Water quality status	Grade
0–25	Excellent	I
25–50	Good	II
50–75	Poor	III
75–100	Very poor	IV

Based on WQI calculation of the samples of 16 locations, they have been graded in Table 11.

**Table 11:** Water quality index (WQI).

Location Code	LAH-01	LAH-02	LAH-03	LAH-04	LAH-05	LAH-06	LAH-07	LAH-08
WQI	45.90	44.57	51.62	47.12	47.39	302.29	44.61	49.25
Grade	II	II	III	II	II	–	II	II
Location code	LAH-09	LAH-10	LAH-11	LAH-12	LAH-13	LAH-14	LAH-15	LAH-16
WQI	41.19	41.07	44.35	54.21	52.36	58.47	53.36	44.56
Grade	II	II	II	III	III	III	III	II

## Results

The use of data visualization techniques, ML and statistical computing has produced some significant results about Lahore water ensemble. An anomalous concentration parametric value of TDS was pointed out by BOXplot method in the location LAH-13. The graphical display of XBAR chart distinguished between the variables showing higher tendency to interact with environmental components and the variables with minimal interaction proclivity. The variables EC, CL and TDS were placed in the band of variability between 0.31–0.62 for demonstrating the higher correlation. In order to seek the locations/samples displaying far-off water elements’ concentration, an algorithm ML-pca was used. The application of the computing method highlight the samples collected from location LAH-06, LAH-10, LAH-13 and LAH-14 for further assessment of components contaminating the water. Moreover, the task of marking down a higher dimensionality data was achieved by setting a variability tolerance bound through an ML-factoran scheme. In order to determine the clusters’ configuration of 18 predictor variables and to identify their domains, a synthesis of cophenetic coefficient and stem algorithm was employed. Clustering of variables conducted using a combination of ML schemes presented an efficient way to establish a grouping of variables, exerting an aggregate influence on an aquatic system.

## Discussion

An incisive combination of ML schemes and statistical methods has been chosen to gauge current water standing of Lahore. These methods are tested on data regarding concentration values of water parameters in the samples collected from 16 strategic locations of the city. A categorical analysis has been presented concerning employment of specific methods and their potential to identify significant features of the data. This study emphasizes on dexterity of these methods^5,12^ to include or exclude some variables to prevent overabundance. The demonstration of quantitative results through application of statistical disposition is administered alongside enumeration of machine learning tools supporting the course of their attainment. Each method’s role in calling attention to some definitive character of collected water samples has been subsequently discussed.

The Boxplot (Fig. 3) of water components’ concentration assisted at the outset of any specific method’s application in identifying higher (EC & TDS) and lower ( $HCO_3$ & Hard) variability parameters throughout the data. Using this display of distribution of the data, we determined an outlier water component in addition to distinguishing the inconsistent variables^2^. It was demonstrated that the sample collected from location LAH-13 contained out of normal range TDS concentration. A seemingly interminable table of pairwise correlations was obtained but an efficient display of peaked and based correlations through XBAR chart (Fig. 4) allowed to circumscribe the set of variables contributing to minimal/maximal pairwise interaction. An XBAR control insight can be utilized to renounce the least correlated variables such as pH, As, Total Coliforms and E. Coli. This approach is further supported by the compass diagrams (Figs. 7 and 8) of Total Coliforms and E. Coli where their modicum correlations with other variables are reflected through higher concentration on $180^{\circ }$ (showing negative correlations) in the circular strip between −0.2 and 0. In an equivalent manner, XBAR chart efficiently picked the highest correlated variables EC, Cl and TDS. Furthermore, the compass diagrams (Figs. 5 and 6) of EC and TDS veritably provided that 61% of their pairwise correlation values are attained in the range of 0.5 and 1. The knowledge of the highest and least correlated variables can be used before conduction of principal component analysis and factor analysis for better deterministic results^10^.

By virtue of the components plot (Fig. 9), obtained through principal component analysis (PCA), four locations LAH-06, LAH-10, LAH-13 and LAH-14 were objectified for extreme propensity. An observation through the data illustrated an out of normal range concentration of one or more water components’ in the samples collected from these locations. The occupation of 1.05mg/l Fe (normal: 0.30mg/l), 266mg/l $SO_4$ (comparatively higher), 1103 mg/l TDS (normal: 1000 mg/l) and 470 mg/l $HCO_3$ (comparatively higher) was noted in water samples from locations LAH-06, LAH-10, LAH-13 and LAH-14, respectively. An output of PCA ‘explained’ assisted in calculating the percent variance elucidated by each principal component. A Scree plot (Fig. 10) of these percent variances records that only first four components can contribute to 95% of the variances of the whole data. The graph (Fig. 11) of first two outputs of PCA projected onto first two principal components demonstrates the contribution of variables EC, TDS, Mg and Cl to the first component owing to their highest coefficient values. This graph also points to the fact that second principal component classifies two sets of variables with positive (Fe, Turbidity Ca, Hard, Mg, Cl, K) and negative ($NO_3$, $HCO_3$, EC, TDS, $SO_4$, Na, F) coefficients. The information provided by this graph can be utilized to classify huge data into smaller groups to study the influence of their aggregate variability on the whole system. The use of PCA in the assessment of environmental data was established to bring out accurate and substantial results^5,12,18,34,36^. However, the integration of ML-pca into our analysis has increased the computing efficiency of the problem as weighted coefficient and components of latent and t-squared elements were taken into account concurrently by the algorithm.

Factor Analysis (FA) has supplied substantial knowledge regarding interdependence of groups of variables that can be directed towards pairing up certain components to investigate their fused influence. An output of FA (Table 5) obtained through computational program factoran has warranted axing of some variables with specific variance below 0.005. It can be observed from specific variance figures that only the variable Mg shows higher independent variability i.e. 0.132. The potential of FA to analyze given data through 5 common factors was tested through* p* value. The obtained value $p=0.1916$ (away from zero) asserted the use of five common factors in making an accurate analysis. Another significant output of FA, rotated estimates of loadings (coefficients of variables expressed in 5 common factors), determined which variables were mainly described by which one of the five factors. It is ascertained through Fig. 12 that variables EC, Mg & K; Turbidity & Fe; Cl & F; $SO_4$ & $NO_3$ and Ca are explained by first (purple), second (blue), third (green), fourth (brown) and fifth (yellow) factors, respectively. This fact is further corroborated by the highest rotated loading values (highlighted in Table 6) of variables against corresponding factors. Moreover, to strengthen the notion that each variable is affected by only one common factor, plots of rotated factor scores and rotated factor loadings were obtained. Figs. 13 and  14 evidently displayed the variables (indicated in numbers) in the direction of respective factors along with their corresponding coefficient values. Some scholarly contributions^10,35,37^ emphasized on using rotated estimates of loadings for factor analysis of a multivariate data, however, we have determined that a strategic exclusion of some variables using specific variance bound (facilitated by ML-factoran) before rotated estimates of loadings computation can accelerate and ease the computing process by a substantial margin^26,31,38,40^.

By means of hierarchical clustering, clusters of variables were compiled in a dendrogram (Fig. 15) with different levels, each level containing similar characteristics’ variables in the form of a group/cluster. An output of agglomerative cluster analysis (CA), pdist, furnished a matrix of distances between every two pairs of variables through an application of machine learning program clusterdata. A linkage function was then employed to create and assign a unique cluster index to a pair of variables/clusters. To validate the cluster division obtained through linkage in Fig. 15, a cophenetic coefficient measuring the correlation between dissimilarity matrix and linkage matrix was obtained. The higher value of an obtained cophenetic coefficient, c = 0.9582 (closer to 1) shows the dendogram to be an accurate reflection of the current water variables data. An inconsistency measure, to compare the heights of links in the dendrogram, was obtained in Table 9. It can be observed that the base links, also called the leaf nodes, connecting the pairs $NO_3$ (12) & F (15), Mg (6) & Cl (8), Ca (5) & As (16) and Na (9) & $SO_4$ will have inconsistency measure as zero due to having no links below them. A higher value of an inconsistency coefficient shows that the corresponding link joins the highly distinct variables or clusters. The use of inconsistency coefficient was also capitalized by creating the inconsistent links’ boundaries through a cutoff inconsistent measure 0.7842. This cutoff assisted in classifying all variables into 7 clusters, each cluster containing variables of same height in Stem diagram (Fig. 16). The computing potential of one of the unsupervised machine learning methods (K-means Clustering and Hierarchical Clustering) to determine characteristic divide between variables of a high dimensional data has also been corroborated by^30^.

To determine the quality of water samples collected from sixteen locations of the city according to^4^ categorical grades, Water quality index (WQI) was computed and presented in Table 11. The designation of water from locations LAH-01, LAH-02, LAH-04, LAH-05, LAH-07, LAH-08, LAH-09, LAH-10, LAH-11, LAH-16; LAH-03, LAH-12, LAH-13, LAH-14, LAH-15 as grades II & III, respectively is discerned from the table. It can also be viewed that the quality of water at location LAH-06 is beyond poor due to the count $WQI>100$. It is noted that this poor quality is caused due to abnormally high iron count of 1.05 mg/l (exceeding normal limit of 0.3 mg/l) prevalent in the water. Although higher iron consolidation in water does not directly pose a health risk but its excessive absorption can lead to futility of the efforts made to eliminate the bacteria due to its rapid interaction with other water components^28^.

### Comparative significance chart

A comparative chart demonstrating the significant features of currently employed collusion of statistical methods and ML schemes has been presented through the Table 12.

**Table 12:** Comparison of current schemes with literature methods.

Current model	Contemporary model/s
Identification of anomalous concentration parametric values before application of inference methods through data visualization	Direct application and analysis of concentration parametric values
Integration of information about anomalous concentration parameters in statistical methods and ML schemes	Assessment of data through direct application of statistical computing
Visual display of higher correlation variables in a certain band of variability	Display of computational values of correlation of variables in a table
Identification of contaminant samples’ locations using collusion of ML-pca and XBAR	Identification of contaminant samples’ locations with individual employment of pca or ML-pca
Dimensionality mark down through an ML-factoran scheme	Application of factoran without tolerance bound setting
Application of synthesizing cophenetic coefficient and stem algorithm for clusterdata	Direct application of clusterdata

## Limitations of the study and future research directions

Some limitations of the present study and future research directions have been highlighted here.

- A high-powered computing infrastructure is required to run ML algorithms of the study.
- Before implementation of statistical and machine learning schemes on water components ensemble, the data was organized and normalized. The application of these algorithms on a non-normalized data can render different results.
- A factor of dimension denouncement due to approximately zero variability of parameters was incorporated in the study. In view of this aspect of the analysis, predictive performance of machine learning schemes can be expected to be a little far-off hundred percent.
- The scope of present results can be broadened by including more parametric components and samples.
- A dual validation of present results can be sought by eliminating the least correlated variables altogether from the data and comparing the obtained results on a new data with present outcomes.
- In order to assess and enhance the performance accuracy of collusion of schemes, the present data can be used for training a neural network in deep learning analysis to study a completely different environmental data.
- A conduct of state of the art (SOTA) analysis shall be performed in future to ensure the performance accuracy of synthesis of machine learning schemes and statistical analysis methods. A prototype of the modelled structure and schemes can be created to study other models in environmental and medical science.
- An approach of ensemble learning can be applied for evaluation of water ensemble, and can be compared with present outcomes for accuracy assessment.

## Conclusion

A categorical analysis of a coarse water-concentration data has been conducted through consolidation of inference methods and ML schemes. An approach of building a spectrum of colluding results highlighting certain features of the data has been experimented to assess comprehensive water stature. The Boxplot of water components’ concentration assisted in highlighting the location LAH-13 that contained out of normal range TDS concentration. The classification of lower and higher variability parameters carried out by XBAR control identified a set of least correlated variables such as pH, As, Total Coliforms and E. Coli. The use of Boxplot and XBAR has been underscored in the analysis before application of statistical and machine learning methods to meticulously watch the outlier water component and its influence on the aggregate system. The information about mutual variability of parameters in addition to their specific variance was integrated in the dimension rebate for ML algorithm factoran application. The use of PCA to study aggregate variability of parameters and to identify them based on normal substance congregation provided four locations LAH-06, LAH-10, LAH-13 and LAH-14 for extreme concentration propensity. The conduction of ML-PCA using weighted variables does not only reveal critical samples from among 16 selected locations, it provides a categorization of variables supported by components of PCA. The knowledge of ML-PCA was synthesized with unsupervised machine learning clustering method to yield an accurate parameters’ centralization. An execution of FA through an ML program factoran demonstrated that a specific tolerance of independent variability ‘0.005’ could be employed to reduce dimension of a system without loss of fundamental data information. It is exhibited through the analysis that the by-product of factoran, rotated loading estimates, can be used to provide water components’ percentage in particular aqua samples. A hierarchical clustering scheme configured a dendrogram for clusters of similar characteristics’ variables. The higher value of cophenetic coefficient, c = 0.9582 provided the validation for an accurate cluster division. The execution of stem algorithm yielded a cutoff inconsistent measure 0.7842, efficiently distinguishing huge set of water concentration variables in to 7 clusters based on similarity and consistency criteria. The nesting of information, obtained through a preceding algorithm application, in a consequent algorithm of assessing a data is presented to exhibit the data distribution, clustering divide and categorization of sample based on outlier component occupation with a better computing precision.

A Water Quality Index (WQI) measure was employed to distinguish the locations with samples of compromised water quality in accordance with Brown water allocation grades. The locations LAH-03, LAH-12, LAH-13, LAH-14 and LAH-15 were identified with poor quality type and, the location LAH-06 was marked with beyond-poor status due to hazardous elemental concentration. The current water gradation in accordance with global standards suggests the need of imperative measures to enhance the quality of water in the region.

## Data Availability

The data that supports the findings of this study is available from the corresponding author, [NS], upon reasonable request.
